# Description of a Deformed, Fragmentary Human Skull

**Published:** 1860-01

**Authors:** 


ART. XI.—*Description of a Deformed, Fragmentary Human Skull.* By J. AITKEN MEIGS, M.D., Professor of the Institutes of Medicine in the Medical Department of Pennsylvania College, etc. etc. Philadelphia, 1859.

THIS little brochure is a reprint from the Proceedings of the Academy of Natural Sciences of Philadelphia, before which it was read during the past year. It is an attempt by the learned author, who is well known both here and in Europe as one of our most able and scientific investigators, to determine, by the configuration of the skull alone, the ethnical type to which it belonged. The skull was presented to him by Dr. J. Judson Barclay, of Jerusalem, who discovered it in an ancient quarry-cave in the vicinity of that city. We have not time to follow Professor Meigs in his erudite discussions; we shall, therefore, content ourselves with two short extracts, setting forth his conclusions.

"Thus, then, from the foregoing details, we may conclude quite positively that the skull found by Mr. Barclay is neither that of a Jew, Arab, Egyptian, Fellah, Turk, Roman, Persian, Elamite, Tibarenian, nor Libyan. Reasons have also been adduced opposing the ascription to it of a Peruvian origin.

"It may have belonged to the Parthians, Phrygians, Mesopotamians, Cappadocians, or Cretans, in so far as these are representatives of the so-called Turanian type. The craniographic data necessary to determine this point satisfactorily are almost entirely wanting."

